# Multi-channel deep learning model-based myocardial spatial–temporal morphology feature on cardiac MRI cine images diagnoses the cause of LVH

**DOI:** 10.1186/s13244-023-01401-0

**Published:** 2023-04-24

**Authors:** Kaiyue Diao, Hong-qing Liang, Hong-kun Yin, Ming-jing Yuan, Min Gu, Peng-xin Yu, Sen He, Jiayu Sun, Bin Song, Kang Li, Yong He

**Affiliations:** 1 Department of Radiology, West China Hospital of Sichuan University, Chengdu, Sichuan China; 2 Department of Radiology, First Affiliated Hospital to Army Medical University (Third Military Medical University Southwest Hospital), Chongqing, China; 3 Institute of Advanced Research, Infervision Medical Technology Co., Ltd, Beijing, China; 4 Department of Radiology, Yongchuan Hospital, Chongqing Medical University, Chongqing, China; 5 Department of Radiology, Chongqing General Hospital, University of Chinese Academy of Sciences, Chongqing, China; 6 Department of Cardiology, West China Hospital of Sichuan University, 37 Guo Xue Xiang, Chengdu, 610041 Sichuan China; 7 Department of Radiology, Sanya Municipal People’s Hospital, Sanya, Hainan China; 8 West China Biomedical Big Data Center, Med-X Center for Informatics, West China Hospital, Sichuan University, 37 Guo Xue Xiang, Chengdu, 610041 Sichuan China; 9 Med-X Center for Informatics, Sichuan University, Chengdu, China

**Keywords:** Cardiac cine MRI, Left ventricular hypertrophy, Case prediction, Deep learning

## Abstract

**Background:**

To develop a fully automatic framework for the diagnosis of cause for left ventricular hypertrophy (LVH) via cardiac cine images.

**Methods:**

A total of 302 LVH patients with cine MRI images were recruited as the primary cohort. Another 53 LVH patients prospectively collected or from multi-centers were used as the external test dataset. Different models based on the cardiac regions (Model 1), segmented ventricle (Model 2) and ventricle mask (Model 3) were constructed. The diagnostic performance was accessed by the confusion matrix with respect to overall accuracy. The capability of the predictive models for binary classification of cardiac amyloidosis (CA), hypertrophic cardiomyopathy (HCM) or hypertensive heart disease (HHD) were also evaluated. Additionally, the diagnostic performance of best Model was compared with that of 7 radiologists/cardiologists.

**Results:**

Model 3 showed the best performance with an overall classification accuracy up to 77.4% in the external test datasets. On the subtasks for identifying CA, HCM or HHD only, Model 3 also achieved the best performance with AUCs yielding 0.895–0.980, 0.879–0.984 and 0.848–0.983 in the validation, internal test and external test datasets, respectively. The deep learning model showed non-inferior diagnostic capability to the cardiovascular imaging expert and outperformed other radiologists/cardiologists.

**Conclusion:**

The combined model based on the mask of left ventricular segmented from multi-sequences cine MR images shows favorable and robust performance in diagnosing the cause of left ventricular hypertrophy, which could be served as a noninvasive tool and help clinical decision.

**Supplementary Information:**

The online version contains supplementary material available at 10.1186/s13244-023-01401-0.

## Key Points

1. Multi-view cine images provide a potential etiology diagnosis tool for LVH.
2. AI-based myocardium spatial–temporal features extracted from images is helpful in diagnosis.
3. AI demonstrated higher accuracy and greater robustness with human experience added.

**Supplementary Information:**

The online version contains supplementary material available at 10.1186/s13244-023-01401-0.

## Introduction

Early and accurate recognizing the etiology of left ventricular hypertrophy (LVH) is key to downstream clinical management and prognosis prediction [1, 2]. Hypertrophic cardiomyopathy (HCM), cardiac amyloidosis (CA) and hypertensive heart disease (HHD) are the common etiologies for LVH while differentiation diagnosis among them can be difficult [3]. Currently, cardiac magnetic resonance imaging (MRI) is one of the important imaging modalities in the work up for LVH etiology classification [4, 5]. A successful, thorough and multi-sequence enhanced MRI examination can help differentiate HCM, or CA from each other, but cardiovascular imaging expert and experience is required [6]. Furthermore, features such as reduced diastolic function, global strain, and presence of late Gadolinium enhancement (LGE) can be found in all of the three diseases. These significant overlap clues and deficiency of definite criteria make the etiology diagnosis even harder. In addition, mapping sequences would usually require additional post-processing analysis using commercial software or experienced cardiovascular imaging doctors to achieve a correct interpretation [7]. Thus, a rapid, simple and noninvasive tool is surely desired.

Artificial intelligence (AI) brew new life into medicine during recent years [8, 9]. The very complex heart structure has spawned numerous innovations in artificial intelligence in this area [10, 11]. Deep learning (DL)-based disease diagnosis algorithm has proved superb ability in diagnosis of arrhythmia, recognition of myocardial infarction lesion and prediction of coronary artery disease [12–14]. Cine images play a basic and key role in heart disease diagnosis as it provides both direct observation and accurate quantification of morphology and function features [15]. Thus, cine-based AI model can potentially serve as an early and rapid screening tool to triage patients with high suspicion of HCM or CA. However, past cardiac AI models are rarely based on cine images. Possible reason might be the large amount of human labor needed for drawing region of interest (ROI) on cine images since segmentation is the first and often the most labor-intensive step [16]. In addition, how to borrow the diagnostic logics from doctors by extracting both the three-dimensional based spatial information and cardiac motion information from the cine images remains a challenge.

Thus, we sought to develop a DL-based fully automatic framework for myocardium segmentation and etiology diagnosis for patients diagnosed with LVH on multi-view cine MRI images through retrospectively collected data. The performance of the final model on diagnosis efficiency would be validated through another prospectively collected independent testing cohort and compared against human radiologists and cardiologist.

## Materials and methods

This study was approved by the center’s Biomedical Research Ethics Committee. Informed consent of retrospectively included data was waived and those of prospective collected data were acquired at the corresponding hospital.

### Patient enrollment

The patients used as primary cohort in this study were retrospectively collected from January 2014 through January 2021. Another dataset used for external testing were prospectively collected from the same center and another three tertiary hospitals. All the cine images were performed as part of the routine cardiac MRI for patients referred to this examination (Details in Additional file 1: Supplemental methods).

For all the included cases, the inclusion criteria were (1) adult patients (age ≥ 18 years), (2) patients with a diagnosis of LVH (defined as with the diastolic wall thickness of at least one segment ≥ 13 mm), and (3) with noted clinical diagnosis based on established diagnostic criteria and MRI measurements of HCM, CA and HHD were included (Details in Additional file 1: Supplemental methods) [4, 17–19]. Those (1) whose diagnosis cannot be confirmed or didn’t belong to the above three categories, (2) whose image data is unavailable for analysis or with poor quality for cine images (3) which were replicated or follow-up examination (namely, one patient had only one examination for this study) were excluded.

**Fig. 1:**
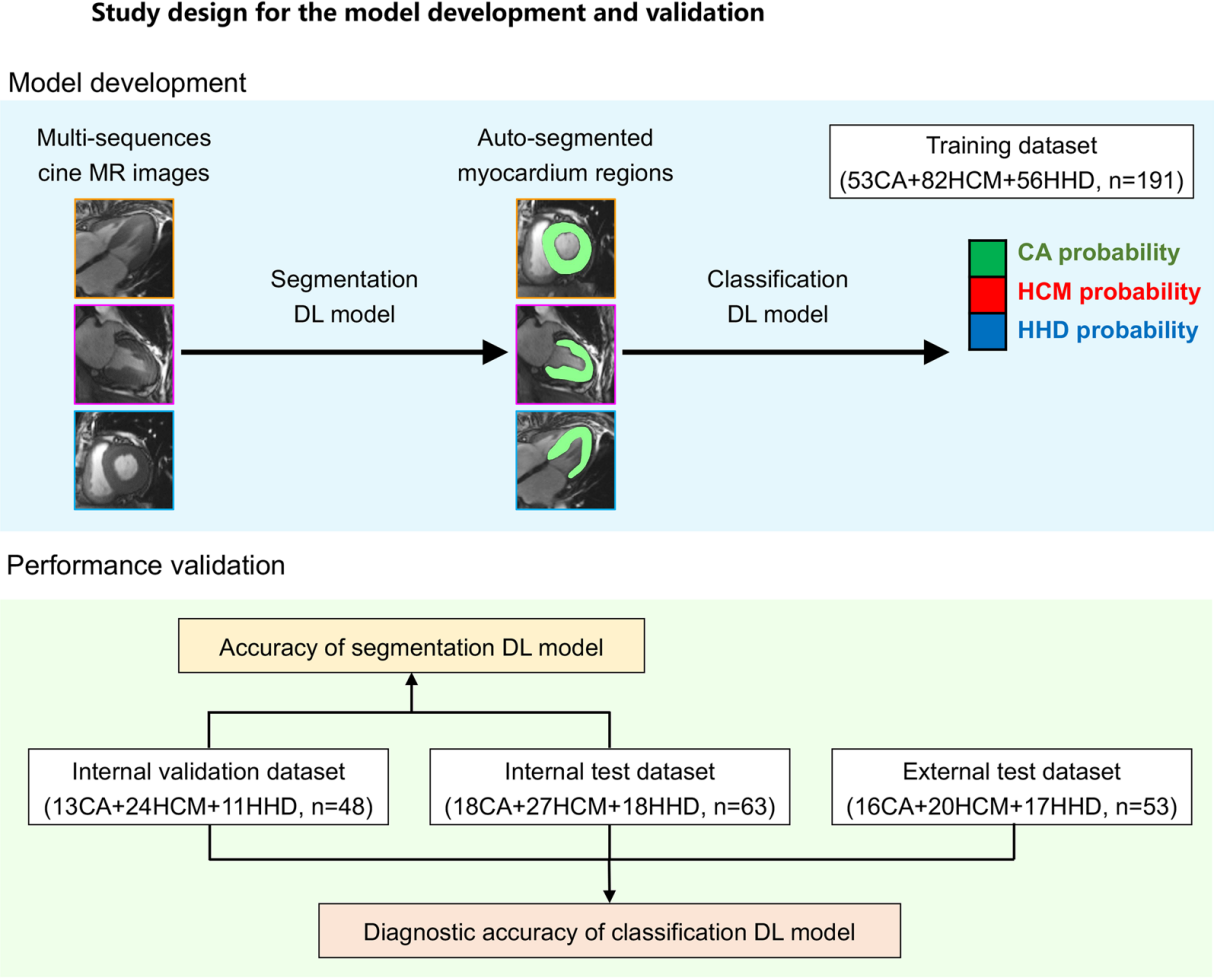
Patient enrollment and study design. HCM—hypertrophic cardiomyopathy, CA—cardiac amyloidosis, HHD—hypertensive heart disease, DL—deep learning

Ultimately, a total of 302 LVH patients from our center were used as primary cohort and were randomly divided into the training (53 CA, 82 HCM and 56 HHD, *n* = 191), validation (13 CA, 24 HCM and 11 HHD, *n* = 48) and internal test (18 CA, 27 HCM and 18 HHD, *n* = 63) datasets. In addition, 22 LVH patients prospectively from the same center and 31 LVH patients from another three hospitals were used as the external test dataset (16 CA, 20 HCM and 17 HHD, *n* = 53) (Fig. 1).

### Left ventricular myocardium segmentation

The open-source software ITK-SNAP (3.8.0, www.itksnap.org) was used for the myocardium segmentation. ROIs containing the left ventricular (LV) myocardium from the two-chamber (2CH), four-chamber (4CH) and one short axis (SAX) at the middle LV level cine images were manually labeled by a radiologist with over five years of experience. The segmented ROIs were confirmed and modified by another senior radiologist with over ten years of experience. Both radiologists were blind to the clinical information and imaging diagnosis report.

### Development of the DL models

**Fig. 2:**
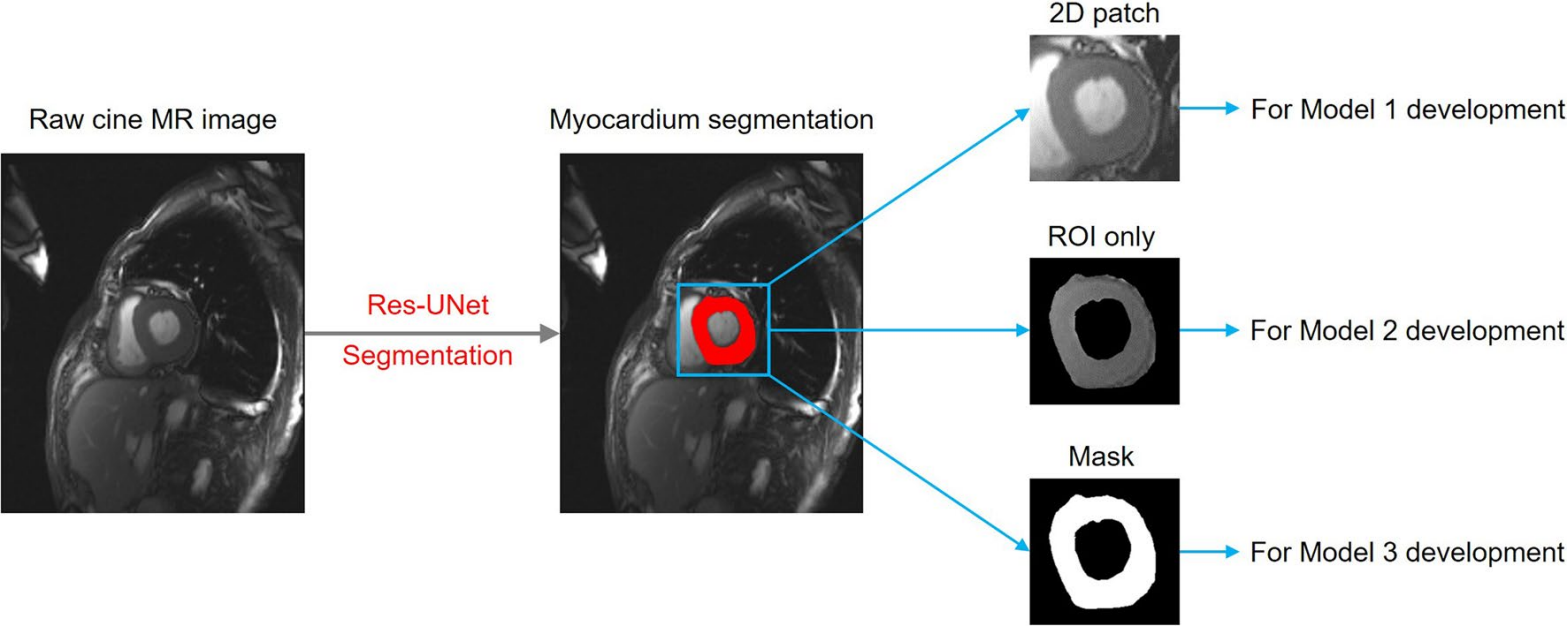
Illustration of data pretreatment for the development of Model 1, Model 2 and Model 3. ROI—region of interest

Reconstruction was performed before model development and all the cineMR images were reconstructed to a resolution of 1 mm × 1 mm × 1 mm. We first developed a modified 2D Res-Unet model to automatically segment myocardium region from cineMR images (Additional file 1). Since there were barely image changes beyond the heart area in cineMR images, we only focused on investigating the myocardium regions in this study. Based on the myocardium that segmented by the Res-Unet model, three classification model were proposed: the model based on the automatic generated 2D patch containing the myocardium regions (Model 1), the model based on the ROI (Model 2), and the model based on the segmentation mask (Model 3). The size of input images was 96 × 96 pixels for all models, which was based on the size of the largest ROI. The exemplar of the automatic generated input images for Model 1, Model 2 and Model 3 is shown in Fig. 2.

**Fig. 3:**
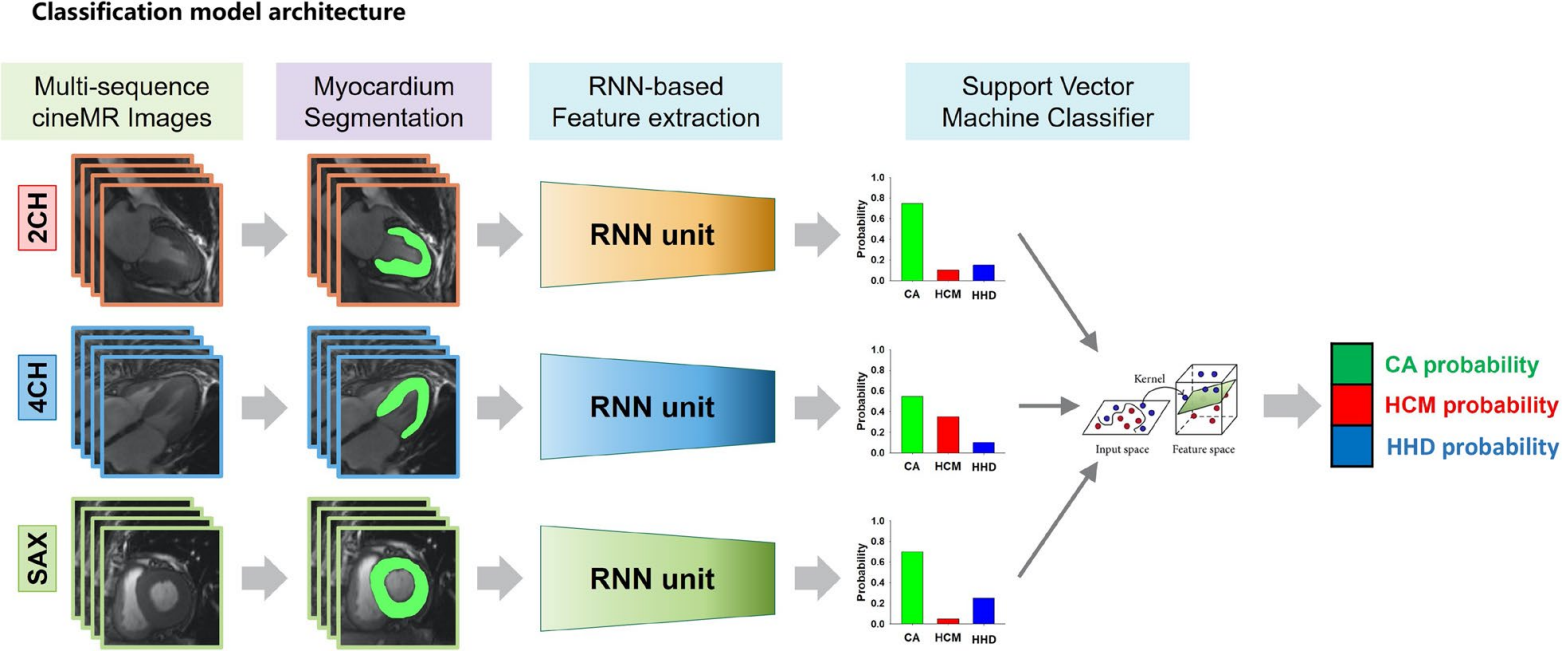
Schematic of the fully automatic framework for predicting the cause of left ventricular hypertrophy through cine MR images. 2CH—two-chamber, 4CH—four-chamber, SAX—short axis

In order to incorporate the series of time-dependent slices from the 2CH, 4CH and SAX sequences of cine MR images, a multi-channel RNN model was proposed to predict the cause of LVH. The convolutional long-short time memory (ConvLSTM) unit was used to deal with the spatial–temporal features extracted from the images in the time-dependent slices (Additional file 1). Finally, a support vector machine classifier was used to integrate the predicted results from multi-sequence cine MR images. The working flow of the fully automatic classification framework is presented in Fig. 3.

The details of model performance evaluation were presented in the Additional file 1.

### Performance comparison between artificial intelligence and the radiologists/cardiologists

The external test dataset was then used for the comparison of diagnostic performance between the best AI model and radiologists/cardiologists. Three radiologists (1 senior with over 10 years of experience, 2 juniors with 3 years of experience), three cardiologists (1 senior with over 10 years of experience, 2 juniors with 3 years of experience) and a cardiovascular imaging expert with more than 10 years of experience were recruited. All radiologists/cardiologists were aware that the patients suffered from LVH, but they were blind to the diagnostic report.

### Statistical analysis

The differences of continuous variables were evaluated through the student’s *t*-test or Mann–Whitney U test, where appropriate. The categorical variables and the comparison of accuracies were evaluated with the Chi-square test. The three-class diagnostic performance was assessed by Cohen’s kappa. Speed of the DL model for diagnosing the cause of LVH was recorded as reported as the mean duration for each case with standard deviation.

The difference between two AUCs of different models were assessed by using Delong’s test [20]. A *P* value less than 0.05 was considered statistically significant. Statistical analysis was performed with R project (v. 3.3.1), the SPSS software (version 23.0) and MedCalc software (version 20.0).

## Results

### Patient characteristics

The mean age for the cases used in training, validation and internal test was 53.3 ± 14.0 years, composed of 181 males and 121 females. The mean age for the included patients of external testing group was 54.6 ± 13.5 years, composed of 31 males and 22 females. There were no significant differences in age (*p* = 0.52) or gender (*p* = 0.84) between the internal and external patients. The HCM cohort consisted of 63/133 (47.4%) cases with hypertrophic obstructive cardiomyopathy, 21/133 (15.8%) cases with apical hypertrophy, and 49/133 (36.8%) cases with other types. The CA cohort consisted of 82/84 (97.6%) cases with light chains, 1/84 (1.2%) case with serum amyloid A and 1/84 (1.2%) cases with transthyretin.

### Automated myocardium segmentation

As shown in Table 1, The Res-Unet model showed favorable myocardium segmentation performance in the validation dataset. The robustness of the Res-Unet model was also confirmed in the internal test dataset. The detailed description of DSCs and HDs were presented in the Additional file 1: Supplemental results.

**Table 1:** Detailed performance of the Res-Unet model in the validation and internal test dataset

	Cine MRI	Validation dataset (*N* = 48)		Internal test dataset (*N* = 63)	
	sequence	Dice (Mean ± SD)	HD (mm, Mean ± SD)	Dice (Mean ± SD)	HD (mm, Mean ± SD)
Per-slice level	2CH	0.934 ± 0.033	2.919 ± 1.740	0.921 ± 0.124	3.111 ± 2.506
	4CH	0.933 ± 0.044	2.975 ± 2.254	0.944 ± 0.037	2.441 ± 2.009
	SAX	0.941 ± 0.040	2.470 ± 2.046	0.944 ± 0.043	2.087 ± 1.141
Per-case level	2CH	0.935 ± 0.028	3.735 ± 1.476	0.921 ± 0.122	4.021 ± 2.077
	4CH	0.934 ± 0.040	4.170 ± 2.286	0.945 ± 0.031	3.531 ± 1.670
	SAX	0.941 ± 0.031	4.510 ± 5.357	0.945 ± 0.031	2.884 ± 1.322

2CH—two-chamber, 4CH—four-chamber, SAX—short axis, HD—Hausdorff distance, SD—standard deviation

Correlation coefficient and Bland–Altman analysis showed high accuracy of the Res-Unet model when compared with human’s performance. (Details in Additional file 1: Supplemental results and Figures S3, S4 and S5).

### Overall accuracy of LVH cause prediction

The speed of the DL model for diagnosing the cause of LVH was very fast, with the average analysis time of roughly 1 s (0.91 ± 0.11, 1.02 ± 0.09 and 0.98 ± 0.10 s in the validation, internal test and external test datasets, respectively).

**Fig. 4:**
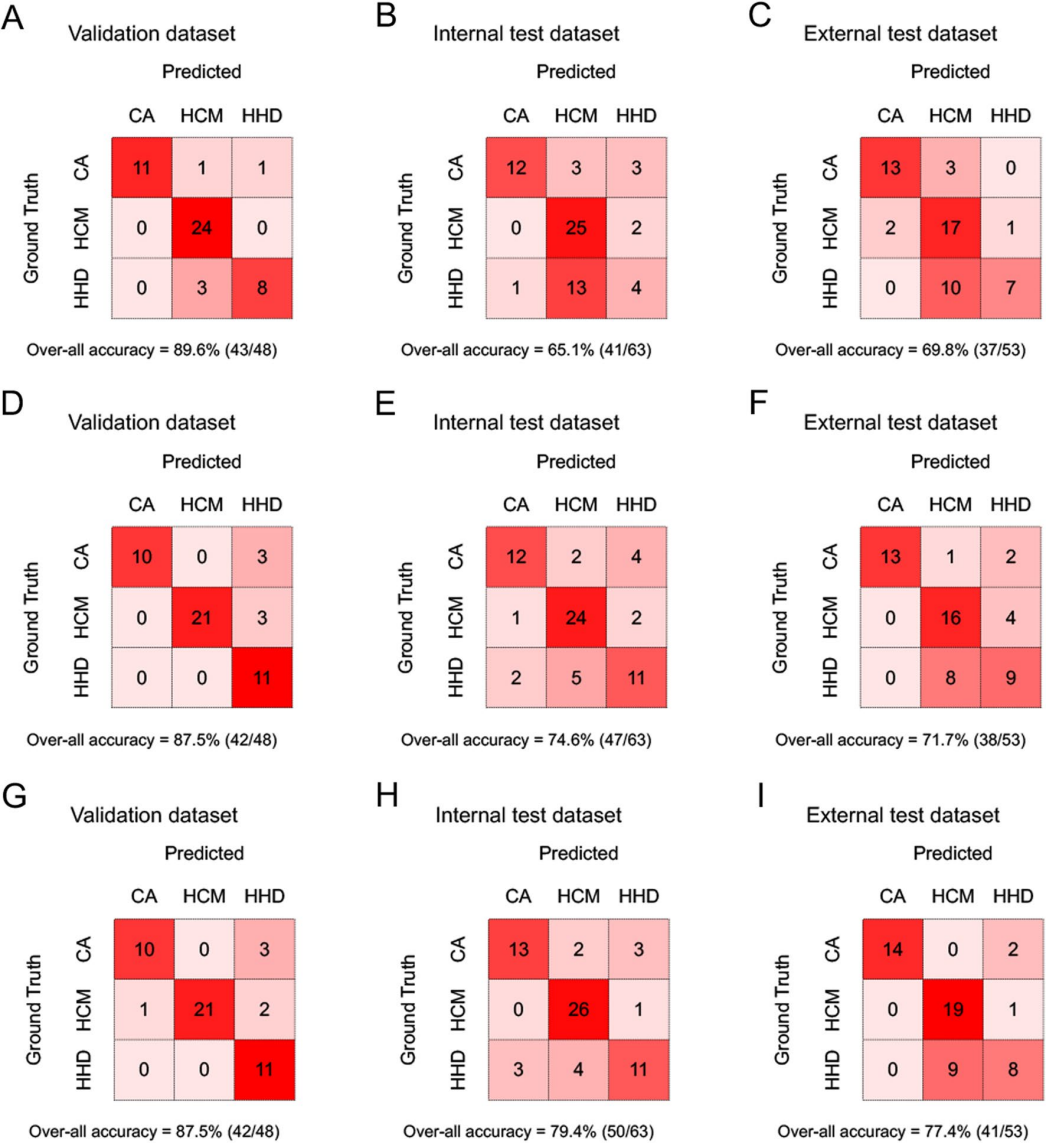
Confusion matrix comparison across Model 1 (**A**–**C**), Model 2 (**D–F**) and Model 3 (**G–I**) in the validation, internal test and external test datasets, respectively. Note that all models noticeably confuse HCM and HHD in the external test dataset. HCM, hypertrophic cardiomyopathy, CA, cardiac amyloidosis, HHD, hypertensive heart disease

All three models showed good classification performance in the validation dataset, with the Cohen’s kappa achieving 0.840, 0.814, and 0.814 for Model 1, Model 2 and Model 3, respectively (Fig. 4 A-C). It seemed that Model 1 suffered from overfitting as the kappa decreased to 0.539 and 0.604 in the internal and external test datasets, respectively. On the contrast, Model 2 and Model 3 exhibited more robust classification performance. The kappa of Model 2 was 0.653 and 0.626 in the internal and external datasets, respectively. The performance of Model 3 was further improved with the kappa up to 0.711 and 0.693 in the internal and external test datasets, respectively. However, the differences in the three-class accuracy of Model 3 compared to other two models were not statistically significant (Chi-square test, *p* = 0.073 vs Model 1 and *p* = 0.526 vs Model 2 in the internal test dataset, *p* = 0.378 vs Model 1 and *p* = 0.504 vs Model 2 in the external test dataset).

The detailed results for comparison of binary classification performance for CA, HCM or HHD across different models were presented in Additional file 1: Supplemental results.

### Comparison between the DL model 3 and radiologists/cardiologists in the external test dataset

**Fig. 5:**
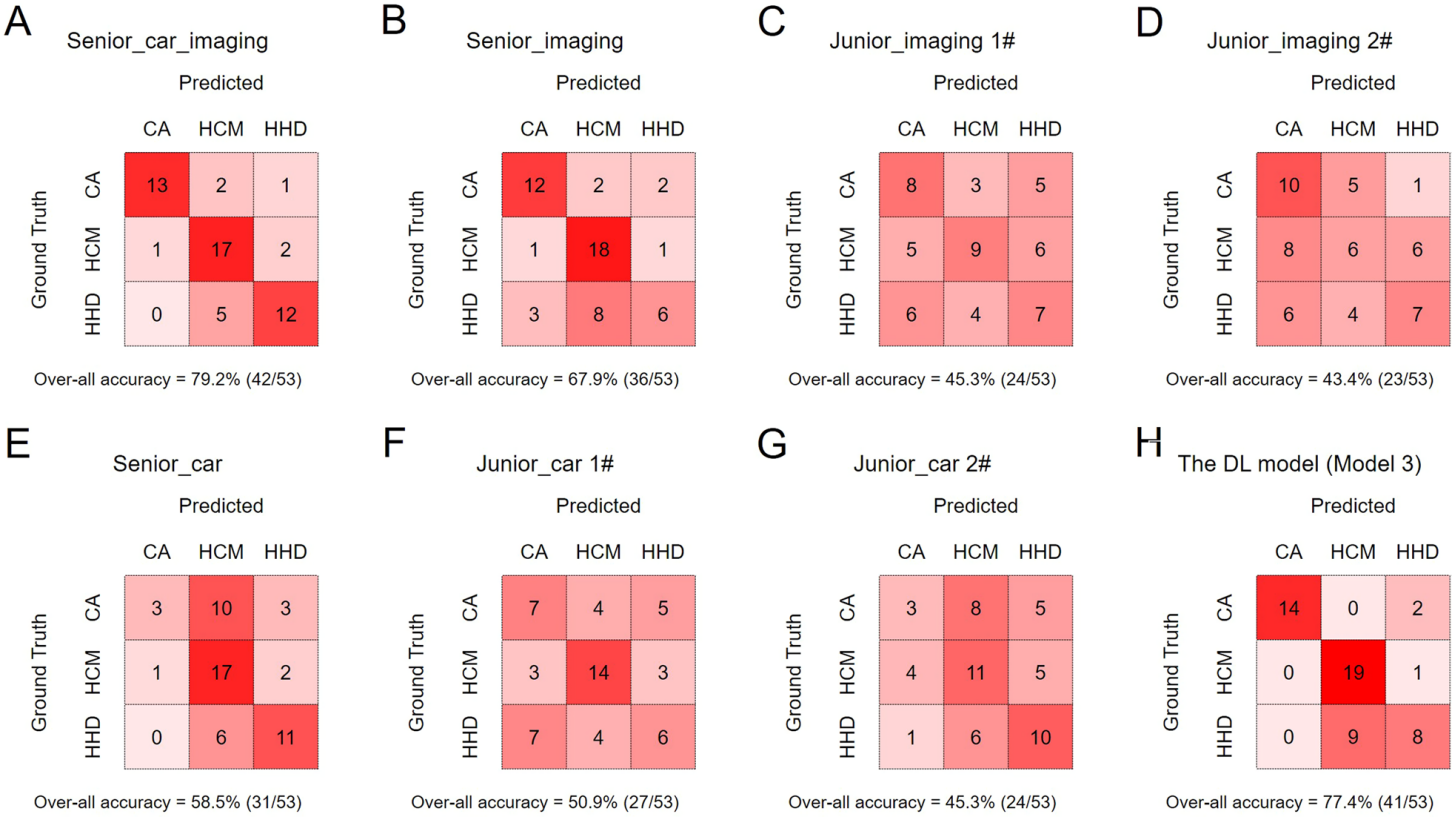
Comparision of the confusion matrix between the senior cardiovascular imaging cardiologist (**A**), senior radiologist (**B**), junior radiologist 1 (**C**), junior radiologist 2 (**D**), senior general cardiologist (**E**), junior cardiologist 1 (**F**), junior cardiologist 2 (**G**) and the Model 3 (**H**) in the external test dataset

The performance of Model 3 was used to compare against that of radiologists/cardiologists in the external test dataset (Table 2). Chi-square analysis indicated that the overall classification accuracy of Model 3 was almost equivalent with the expert cardiovascular radiologist (*p* = 0.814), and outperformed the other doctors (all *p* < 0.05) except the senior radiologist 1 (*p* = 0.276). The individual observer variability analysis indicated that the agreement was moderate for the Model 3 compared with the expert cardiovascular radiologist (kappa = 0.552) and the senior radiologist (kappa = 0.502), while the agreement between the Model 3 and other radiologists/cardiologists was poor (all kappa < 0.2). The confusion matrix of the DL model and the radiologists/cardiologists is shown in Fig. 5.

**Table 2:** Three-class diagnostic performance of the Model 3 and radiologists/cardiologists in the external test dataset

	Three-class Cohen’s kappa	Three-class overall accuracy	*p* value (*vs* model 3)	Reader agreement (*vs* model 3)
DL model 3	0.693	77.4% (41/53)	*Reference*	*Reference*
Expert cardiovascular radiologist	0.717	79.2% (42/53)	*0.814*	*0.552*
Senior radiologist	0.582	67.9% (36/53)	*0.276*	*0.502*
Junior radiologist 1	0.355	45.3% (24/53)	< *0.001*	*0.162*
Junior radiologist 2	0.340	43.4% (23/53)	< *0.001*	*0.163*
Senior cardiologist	0.478	58.5% (31/53)	*0.037*	*0.134*
Junior cardiologist 1	0.405	50.9% (27/53)	*0.005*	*0.147*
Junior cardiologist 2	0.352	45.3% (24/53)	< *0.001*	*0.195*

Italic indicates the results of DL model 3 as the reference, and report if the performance of any other rater (radiologists or cardiologists) had any difference from DL model 3

**Fig. 6:**
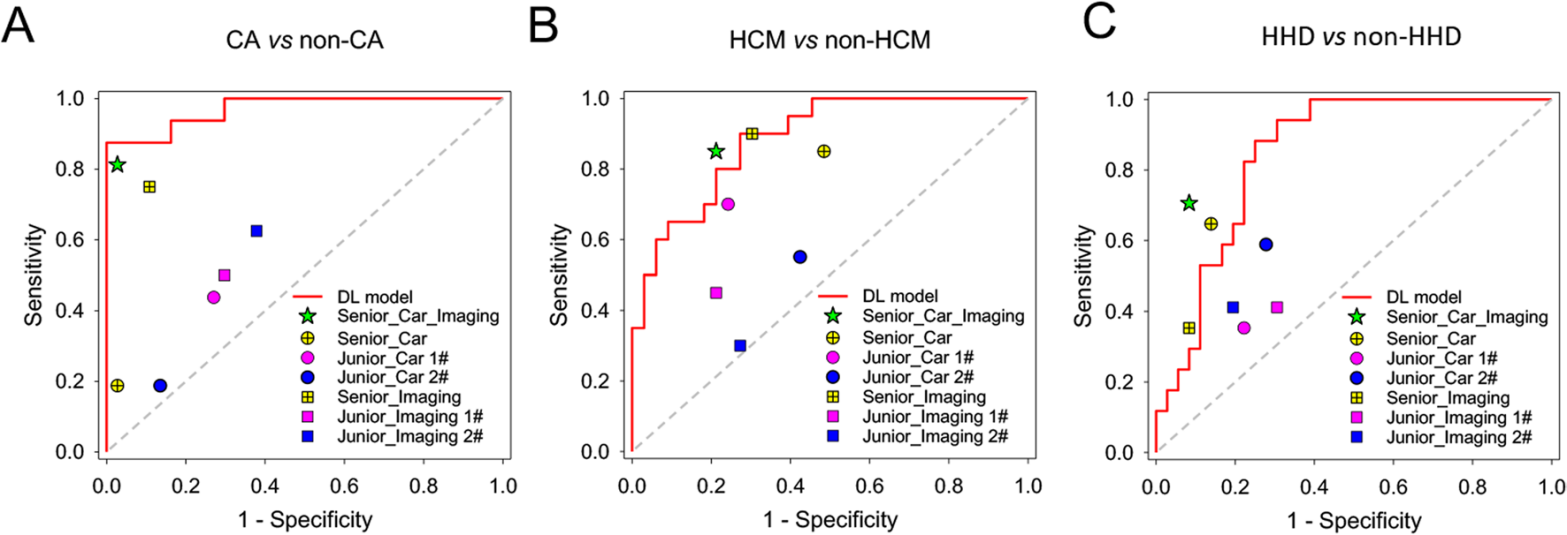
Comparison between the deep learning model (Model 3) and radiologists/cardiologists for the binary classification of CA (**A**), HCM (**B**) or HHD (**C**) in the external test dataset. HCM, hypertrophic cardiomyopathy, CA, cardiac amyloidosis, HHD, hypertensive heart disease, DL, deep learning

Concerning the binary-classification-level comparison (Fig. 6), the AUC of Model 3 was significantly higher than that of the radiologists and cardiologists in discriminating CA from non-CA (all *p* < 0.05), except for the expert cardiovascular radiologist (*p* = 0.190). For the discrimination of HCM from non-HCM, the Model 3 showed non-inferior performance with the expert cardiovascular radiologist (*p* = 0.328), and had achieved higher AUC than other doctors (*p* = 0.127 *vs* senior radiologist, and all *p* < 0.05 *vs* the others). The DL model also showed good performance for the discrimination of HHD from non-HHD, with the AUC competitive with the expert cardiovascular radiologist (*p* = 0.661) and senior cardiologist (*p* = 0.277), and significantly higher than other doctors (all *p* < 0.05). The detailed performance comparison was summarized in Additional file 1: Tables S1 and S2.

## Discussion

In this paper, we introduce a DL-based fully automatically myocardium segmentation and LVH etiology classification AI model working with cine MRI images. The segmentation model achieved robust accuracy in both internal and external validation tests. The diagnostic model achieved cardiovascular imaging expert level and surpassed junior radiologists or cardiac doctors in recognizing LVH etiology in external validation test in a very short time. In addition, comparison among the performances from different subtypes models demonstrated that the DL model with learning based simply on morphology and motion features of the LV myocardium during a cardiac cycle had the highest accuracy and stability. This result supports the idea that training a DL model with human-recognized or acknowledged key features can achieve near-to-expert performance with high efficiency when only limited data is available, and thus help pave the way for further AI research and clinical application in this field.

Our study further clarified the feasibility of cine MRI images in differential diagnosis through DL model for LVH. Zhang et al. developed a chronic myocardial infarction diagnosis DL model on cine MRI with high accuracy by referring to the LGE result, raising the possibility of training DL model to “learn” motion features from cine images [13]. For LVH, numerous studies investigating volume and wall motion parameters have noticed single or combined global morphology and motion indexes can be very valuable in diagnosis of CA and HCM, and HHD [21–23]. Although enormous details in myocardium can now be quantified through meticulous post-processing, it will be very hard for human beings to read and directly interpret such great number of parameters, as shown in the relatively bad performance for the junior doctors. In fact, only global and peak strains are regularly used currently [4, 24]. One of the core competencies of AI compared to human is to extract information from images that is not apparent to the naked eye [25], thus, it is reasonable that DL models developed using cine images can have the capacity of differentiating between these three diseases. In addition, DL model developed in this study worked with a very high speed, which is another inbuilt but nonnegligible merit of AI algorithm. Such advantages made the DL model an ideal fast triage tool for patients suspected with LVH and referred to MRI examination.

Studies have been done to identify LVH through echocardiogram or echocardiography by DL models [26, 27]. Nevertheless, information provided by echo or EKG is limited especially the lack of tissue characterization, which usually provide key factors in further differentia diagnosis or risk stratification for patients with LVH [15, 28]. Study by Khurshid et al. demonstrated the ability of predicting genomic variations through CMR-derived LVMI using DL models. Neisius U’s study demonstrated that radiomic analysis of native T1 images could discriminate HHD from HCM with an accuracy of 80.0% in test sets [29]. Another study by Martini N et al. trained a DL model using LGE images showed accuracy of 88% in detecting CA from other patients [30]. Our study achieved comparable accuracy and added by providing the possibility of morphology-and-motion-feature-based DL model in LVH diagnosis. Combination of morphology, motion and tissue characterization to train DL model may achieve more ideal results and such studies are warranted.

**Fig. 7:**
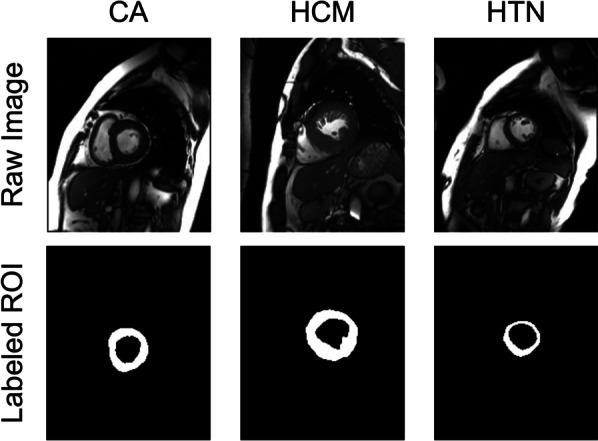
Illustrations of cine images and the corresponding ROIs in Model 3 of different LVH type

Interestingly, when “less” information was put in to train the AI model, outcome turned out to be better. In our study, model 3 outperformed the other two models in both accuracy and robustness. Compared to model 1 and model 2, model 3 was more concentrated on extracting the morphology and motion features by using the mask of the segmented LV myocardium instead of the LV region (model 1) or the myocardium (model 2). Theoretically, MRI process was susceptible to a wide range of artifacts and variability, including but not limited to the manufacturer and scanner, scanning protocol and acquisition parameters. Those artifacts and variability were considerable for the generalization of neural networks (that is, the model 1 and model 2 in this study) on different datasets. On the contrast, the variability of image quality was minimized by the binarization processing of the segmented LV myocardium ROIs in model 3. Contents “learned” to build model 3 is expected to be only the shape of the heart and how this shape is changing throughout the cardiac cycle (Fig. 7). Although the information is simplified, it is also cleaned and purified, which might be the reason for the improved model robustness in the external test dataset. Although the learning process of DL model is like a “black box,” through prespecified information feeding, the training efficiency seems to be improved [31, 32]. Similar comparison could be found in Cao et al.’s study, where the author developed a step-by-step aorta dissection AI segmentation model and proved its superior to the traditionally trained model developed by simple data feeding [33]. As MRI, especially cardiac MRI is relatively time-consuming process and more expensive, resulting in a limited number of data available. Our study contributed by providing a new and efficient way to train the DL model.

Last but not least, our segmentation results were consisted with these previous studies and had achieved higher dice values [34, 35]. We added by updating the architecture in our segmentation model. Compared with other DL-based segmentation models (e.g., Mask R-CNN, FCN and DSN), U-Net was widely used in medical image segmentation and had been proved to be an outstanding network with good performance in small datasets due to the successful combination of low-level and high-level information [36], and the incorporation with ResNet could further improve the efficiency in feature extraction and facilitate more accurately segmentation.

### Limitation

Several limits should be mentioned here. First, limited number of patients were included for this study. However, for this MRI-based study, the number of the data used is comparable to previous published ones and we performed external validation and human-level comparison to further validate our results. Second, LVH manifestation can show up in other diseases including iron-deposition, Anderson-Fabry disease, eosinophilic cardiomyopathy or other inflammatory process in myocardium. In consideration of the number of cases available, we only included three most common etiologies in our center for LVH classification task in this study. Thus, the direct clinical application of the DLAD model developed in this study is limited. Another limitation of our model is the lack of clinical variables or other MRI sequences, which might be also the cause for the relatively unsatisfactory performance of junior cardiologists/radiologists, as they have been used to diagnosing the disease with more information. Especially, whether the performance of such AI model could surpass the diagnostic ability of T1 mapping would require further validation in future studies. Nevertheless, for this study we are trying to build a model based on morphology and motion features extracted from cine images. Further studies with more sequences involved and clinical variables are warranted.

## Conclusion

A fully automatically myocardium segmentation and spatial–temporal morphology feature based LVH etiology diagnosis deep learning framework using cardiac cine MRI was described, with non-inferiority to cardiovascular imaging expert and robust performance in multi-center data. The proposed AI framework could at least facilitate initial LVH etiology diagnosis and potentially, we provided an efficient way to develop a DL model when only limited data is available.

## Supplementary Information

**Additional file 1.** Supplemental methods and results.

## Data Availability

The datasets generated or analyzed during the study are available from the corresponding author on reasonable request.

## Abbreviations

AI: Artificial intelligence
CA: Cardiac amyloidosis
DL: Deep learning
HCM: Hypertrophic cardiomyopathy
LGE: Late gadolinium enhancement
LV: Left ventricular
LVH: Left ventricular hypertrophy
MRI: Magnetic resonance imaging
ROI: Region of interest
