# Supplementary material for: Multi-channel deep learning model-based myocardial spatial–temporal morphology feature on cardiac MRI cine images diagnoses the cause of LVH
Source: Insights Imaging. 2023 Apr 24;14:70. doi: 10.1186/s13244-023-01401-0 (PMC10126185; doi:10.1186/s13244-023-01401-0)
Supplement: Supplementary file 1 — Additional file 1. Supplemental methods and results. [file 13244_2023_1401_MOESM1_ESM.pdf]

ELECTRONIC SUPPLEMENTARY MATERIAL

Multi-channel Deep-Learning model-based myocardial spatial-temporal morphology feature on Cardiac MRI cine images diagnoses the cause of LVH

1. Supplemental methods

1.1. Cine MR image acquisition

For the data used for training and internal test, all the scans were acquired through a 3.0 T MR scanner (MAGNETOM Skyra; Siemens Healthcare). For the images collected from multi-centers outside the major center, another three 3.0 T Siemens MR scanners were used (MAGNETOM Trio a Tim system, SIEMENS Verio Dot, and Skyra).

In detail, Contiguous short-axis (SAX) slices encompassing the whole left ventricle, standard two-, three-, and four-chamber (2CH, 3CH and 4CH) cine images were acquired using Balance Steady state free precession sequence (Repetition time (TR): 39.34 ms; echo time (TE):1.22 msec; flip angle: 40°; Field of view (FOV): 340 x 285 mm2; matrix: 208 x 139; slice thickness: 8mm; and number of phases: 25) during repeated breath holds.

1.2. Detailed inclusion criteria of CA, HCM and HHD for the patients

Cardiac amyloid (CA) was diagnosed based on the combination of typical features on CMR and biopsy proven systemic AL amyloidosis on cardiac or non-cardiac biopsy.

Hypertrophic cardiomyopathy (HCM) was diagnosed in one of two ways: normal LV cavity size with wall thickness ≥15 mm, or a wall thickness above the normal range with high clinical suspicion (i.e., electrocardiogram abnormalities, apical variant phenotype, HCM family history + LVWT ≥13 mm), both not explained by loading conditions [2, 3]. HCM patients with previous septal ablation or myectomy were excluded.

HHD was defined as increased LVWT (≥12 mm) [28] associated with the diagnosis of arterial hypertension [26] in the absence of severe chronic kidney disease, LV cavity dilatation, and

cardiac disease that could result in a similar magnitude of hypertrophy (i.e. moderate-to-severe valvular heart disease, inherited/acquired cardiomyopathies).

1.3. Details in AI model development

1.3.1. Pre-training of the deep learning models

The transfer learning method was applied to improve the robustness and generalization of the deep learning models and to achieve better performance. The neural networks used in this study were first pre-trained on natural images from the ImageNet dataset, and multiple medical image datasets such as the CPTAC-LUAD, CPTAC-LSCC, TCGA-LUAD and TCGA-LUSC datasets from The Cancer Imaging Archive (TCIA) database, and the BraTS dataset from the Kaggle competition (https://www.kaggle.com/awsaf49/brats20-dataset-training-validation) were also used for model pre-training.

1.3.2. Segmentation model development

A modified 2D Res-Unet was used for the segmentation of myocardium regions from the cine MR images. The proposed Res-Unet consisted of an encoder-decoder architecture, which was similar to that of the original U-Net. The encoder part used ResNet-50 backbone for the feature extraction, including one initial sampling module and four residual down-sampling modules. There were four up-sampling modules in the decoder part, and the decoder part contained a skip connection to a residual block. The conceptual architecture of the Res-Unet model was shown in Supplemental Figure 1.

1.3.3. Classification model development

Based on the myocardium that segmented by the Res-Unet model, the fully automated classification model was constructed. Since every patient had 3 sequences of cine MR images (2CH, 4CH and SAX) and each sequence consisted of a series of time-dependent slices, a multi-channel RNN model was proposed for predicting the cause of left ventricular hypertrophy. For each single-channel RNN network, the ResNet-50 network was applied as

the backbone architecture for the feature extraction of each slice, and the convolutional long-short time memory (ConvLSTM) unit was used for learning long sequence dependencies based on time series images. The output of each single-channel RNN was set to a 3-bit vector with each bit contained the probabilities for CA, HCM and HHD, respectively (Supplemental Figure 2). In order to combine the 2CH, 4CH and SAX sequences of cine MR images, a 9-bit vector was generated by fusing the output of three single-channel RNN networks, which consisted of the three 3-bit vectors from the single-channel RNNs based on the 2CH, 4CH and SAX sequences of cine MR images, respectively. A support vector machine (SVM) with sigmoid kernel was used for the final diagnosis. The relevant attribution of each single-sequence cine MR image was evaluated by calculating the weight with respect to the class attribute. The working flow of the fully automatic framework was presented in Figure 3.

Online data augmentation was also applied during the training process of the classification models, including flipping (perpendicular to the x and y axis), random rotation (90, 180, and 270 degrees) and random brightness contrast (80%, 90%, 110% and 120%). The sample size had increased ten times, yielding a total of 1910 samples during model development.

The DL models were constructed and fine-tuned in the training dataset. The DL models were trained based on the binary cross-entropy loss function and the Adam algorithm was applied to optimize the model in the training stage. The mini-batch size, learning rate and weight decay were set to 32, 1×10^{-4} and 0.01, respectively. The dropout rate was set to 0.5 and other parameters were set to their default values. The learn rated would reduce 90% when the model performance did not improve after 15 consecutive epochs, and the training was stopped when the learning rate had decreased to 1×10^{-6}.

The neural networks were implemented using Python 3.6.8 (https://www.python.org) based on the PyTorch deep learning library. The supervised training was performed on a workstation with two GeForce GTX 2070 GPUs (NVIDIA). The training and validation of the

deep learning models were performed on the InferScholar platform (InferVision, version 3.5). The code is open source at https://github.com/smilenaxx/LVH_classification.

1.4. Evaluation of model performance

The accuracy of the segmentation model was analyzed using the Dice similarity coefficient (DSC) and the Hausdorff distance (HD) [22]. The agreement between automated segmented left ventricle volume and corresponding reference values was evaluated by using linear regression and Bland-Altman analyses.

The diagnostic capability of three-class classification was assessed by overall accuracy and Cohen’s kappa coefficient, and the discriminative efficacy of binary classification was evaluated by the receiver operating characteristics (ROC) analysis with respect to the area under the curve (AUC). The sensitivity, specificity, positive predictive value (PPV) and negative predictive value (NPV) were also calculated under the optimal threshold according to the maximum Youden index [23].

2. Supplemental results

2.1. Performance for myocardium segmentation

The Res-Unet model showed high accuracy for myocardium segmentation with DSCs achieved 0.934, 0.933, 0.941 and HDs achieved 2.919 mm, 2.975 mm, 2.470 mm for the 2CH, 4CH and SAX sequence cine MRI images at per-slice level in the validation dataset, respectively. The per-case level DSCs and HDs were 0.935, 0.934, 0.941 and 3.735 mm, 4.170 mm, 4.510 mm for the 2CH, 4CH and SAX sequence cine MRI images, respectively. Similar performance was observed in the internal test dataset, with the DSCs for the 2CH, 4CH and SAX sequence cine MRI images yielding 0.921, 0.944 and 0.944 at per-slice level,

and 0.921, 0.945 and 0.945 at per-case level, respectively. The HDs for the 2CH, 4CH and SAX sequence cine MRI images were 3.111, 2.441 and 2.087 at per-slice level, and 4.021, 3.531 and 2.884 at per-case level, respectively (Table 1).

2.2. Correlation coefficient and Bland-Altman analysis for the segmentation model

Correlation coefficient (R) and Bland-Altman analysis were also applied in the validation and internal test datasets to evaluate the performance and robustness of the Res-Unet model (Supplemental Figures 3 and 4). There were strong correlations between the automatic segmentation results and the manually labeled myocardium volumes at per-case level analysis in both the validation dataset (R^2=0.949, p<0.001 for the 2CH sequence; R^2=0.932, p<0.001 for the 4CH sequence; R^2=0.925, p<0.001 for the SAX sequence) and the external test dataset (R^2=0.918, p<0.001 for the 2CH sequence; R^2=0.945, p<0.001 for the 4CH sequence; R^2=0.915, p<0.001 for the SAX sequence). In Bland-Altman analysis, the automatic segmentation results and the manually labeled myocardium volumes showed good agreement in both the validation and internal test dataset. These results indicated that our Res-Unet model could segment myocardium at high accuracy while maintaining stable performance across different datasets. The typical 2CH, 4CH and SAX segmentation examples of our deep learning model were presented in Supplemental Figure 5.

2.3. Analysis of the attribute weights of the SVM model

The weights of the attributes (i.e., the input images from 2CH, 4CH and SAX sequence of cine MR images) in the SVM model was assessed to make the predictive models more interpretable (Supplemental Figure 6). The SAX sequence images showed the least contribution to the SVM classifier in all three models. Model 2 put the most emphasis on the 4CH sequence images while the greater reliance on the 2CH sequence images was observed in the Model 3.

2.4. Comparison of binary classification performance for CA, HCM or HHD across different models

All models showed almost perfect performance for binary classification of CA, HCM or HHD in the validation dataset, with the AUCs varied from 0.961~1.000 (Supplemental Figure 7 A-C). In the external test dataset, Model 1, Model 2 and Model 3 had achieved AUCs of 0.889 (95% confidence interval (CI), 0.772~0.958), 0.946 (95% CI, 0.847~0.989) and 0.971 (95% CI, 0.884~0.998) for differentiating CA from non-CA, 0.765 (95% CI, 0.629~0.871), 0.855 (95% CI, 0.731~0.936) and 0.885 (95% CI, 0.767~0.956) for differentiating HCM from non-HCM, and 0.786 (95% CI, 0.652~0.887), 0.820 (95% CI, 0.691~0.912) and 0.848 (95% CI, 0.723~0.932) for differentiating HHD from non-HHD, respectively (Supplemental Figure 7 G-I).

Delong's test demonstrated that all models showed almost equivalent performance for the binary classification of CA, HCM or HHD in the validation dataset (all p values > 0.05). Model 2 and Model 3 showed better performance than Model 1 in the internal test dataset, the AUCs of Model 3 were significantly higher than that of Model 1 for the binary classification of HCM (p = 0.001) and HHD (p = 0.045), and Model 2 also outperformed Model 1 in distinguishing HCM from non-HCM (p = 0.016). Similar model performance was observed in the external test dataset, as Model 3 outperformed Model 1 for the binary classification of CA (p = 0.088) and HCM (p = 0.067). (Details in Supplemental Table 2).

3. Supplemental Tables

Supplemental Table 1. Comparison between the deep learning model (Model 3) and radiologists/cardiologists for the diagnosis of CA, HCM and HHD in the external test dataset.

Task	Model/Radiologist	AUC (95% CI)	*p-value*	threshold	sensitivity	specificity	PPV	NPV
**CA vs non-CA**	DL model	0.971 (0.884~0.998)	reference	>0.2401	87.50%	100.00%	100.00%	94.90%
	Senior_Car_Imaging	0.893 (0.777 to 0.961)	0.19	N/A	81.30%	97.30%	92.90%	92.30%
	Senior_Car	0.580 (0.437 to 0.714)	<0.001	N/A	18.80%	97.30%	75%	73.50%
	Junior_Car 1#	0.584 (0.440 to 0.717)	<0.001	N/A	43.80%	73.00%	41.20%	75.00%
	Junior_Car 2#	0.526 (0.384 to 0.665)	<0.001	N/A	18.80%	86.50%	37.50%	71.10%
	Senior_Imaging	0.821 (0.691 to 0.913)	0.029	N/A	75.00%	89.20%	75.00%	89.20%
	Junior_Imaging 1#	0.601 (0.458 to 0.733)	<0.001	N/A	50.00%	70.30%	42.10%	76.50%
	Junior_Imaging 2#	0.623 (0.480 to 0.753)	<0.001	N/A	62.50%	62.20%	41.70%	79.30%
**HCM vs non-HCM**	DL model	0.885 (0.767~0.956)	reference	>0.2488	90.00%	72.70%	66.70%	92.30%
	Senior_Car_Imaging	0.819 (0.689 to 0.911)	0.328	N/A	85.00%	78.80%	70.80%	89.70%
	Senior_Car	0.683 (0.540 to 0.804)	0.009	N/A	85.00%	51.50%	51.50%	85.00%
	Junior_Car 1#	0.729 (0.589 to 0.842)	0.062	N/A	70.00%	75.80%	63.60%	80.60%
	Junior_Car 2#	0.563 (0.420 to 0.699)	0.003	N/A	55.00%	57.60%	44.00%	67.90%
	Senior_Imaging	0.798 (0.666 to 0.896)	0.127	N/A	90.00%	69.70%	64.30%	92.00%
	Junior_Imaging 1#	0.619 (0.475 to 0.749)	0.005	N/A	45.00%	78.80%	56.30%	70.30%
	Junior_Imaging 2#	0.514 (0.372 to 0.653)	<0.001	N/A	30.00%	72.70%	40.00%	63.20%
**HHD vs non-HHD**	DL model	0.848 (0.723~0.932)	reference	>0.1809	94.10%	69.40%	59.30%	96.20%
	Senior_Car_Imaging	0.811 (0.680 to 0.906)	0.661	N/A	70.60%	91.70%	80.00%	86.80%
	Senior_Car	0.754 (0.616 to 0.862)	0.277	N/A	64.70%	86.10%	68.80%	83.80%
	Junior_Car 1#	0.565 (0.422 to 0.701)	0.003	N/A	35.30%	77.80%	42.90%	71.80%
	Junior_Car 2#	0.655 (0.512 to 0.780)	0.041	N/A	58.80%	72.20%	50.00%	78.80%
	Senior_Imaging	0.635 (0.491 to 0.763)	0.006	N/A	35.30%	91.70%	66.70%	75.00%
	Junior_Imaging 1#	0.553 (0.410 to 0.690)	0.001	N/A	41.20%	69.40%	38.90%	71.40%
	Junior_Imaging 2#	0.609 (0.465 to 0.740)	0.008	N/A	41.20%	80.60%	50.00%	74.40%

Supplemental Table 2. Model performance of binary classification tasks for CA, HCM and HHD in the validation, internal test and external test datasets.

Dataset	Task	Model	AUC (95% CI)	*p-value*	threshold	sensitivity	specificity	PPV	NPV
Validation	CA vs non-CA	Model 1	0.967 (0.870~0.997)	*reference*	>0.2083	92.30%	100.00%	100.00%	97.20%
		Model 2	0.980 (0.891~1.000)	*0.617*	>0.0734	100.00%	85.70%	72.20%	100.00%
		Model 3	0.998 (0.922~1.000)	*0.365*	>0.2234	100.00%	97.10%	92.90%	100.00%
	HCM vs non-HCM	Model 1	0.977 (0.887~0.999)	*reference*	>0.6884	91.70%	95.80%	95.70%	92.00%
		Model 2	0.984 (0.898~1.000)	*0.736*	>0.2315	95.80%	91.70%	92.00%	95.70%
		Model 3	1.000 (0.926~1.000)	*0.165*	>0.0625	100.00%	100.00%	100.00%	100.00%
	HHD vs non-HHD	Model 1	0.961 (0.861~0.996)	*reference*	>0.2082	100.00%	81.10%	61.10%	100.00%
		Model 2	0.983 (0.895~1.000)	*0.457*	>0.6864	100.00%	91.90%	78.60%	100.00%
		Model 3	0.998 (0.921~1.000)	*0.13*	>0.6987	100.00%	97.30%	91.70%	100.00%
Internal test	CA vs non-CA	Model 1	0.846 (0.733~0.924)	*reference*	>0.0737	77.80%	86.70%	70.00%	90.70%
		Model 2	0.872 (0.763~0.943)	*0.716*	>0.1929	83.30%	82.20%	65.20%	92.50%
		Model 3	0.895 (0.792~0.958)	*0.148*	>0.0728	88.90%	80.00%	64.00%	94.70%
	HCM vs non-HCM	Model 1	0.853 (0.741~0.930)	*reference*	>0.6884	85.20%	72.20%	69.70%	86.70%
		Model 2	0.953 (0.867~0.990)	*0.016*	>0.6905	96.30%	88.90%	86.70%	97.00%
		Model 3	0.966 (0.887~0.995)	*0.001*	>0.1856	100.00%	77.80%	77.10%	100.00%
	HHD vs non-HHD	Model 1	0.758 (0.634~0.857)	*reference*	>0.2018	77.80%	71.10%	51.90%	88.90%
		Model 2	0.836 (0.721~0.917)	*0.273*	>0.2236	77.80%	86.70%	70.00%	90.70%
		Model 3	0.881 (0.775~0.949)	*0.045*	>0.2031	100.00%	68.90%	56.30%	100.00%
External test	CA vs non-CA	Model 1	0.889 (0.772~0.958)	reference	>0.0716	87.50%	89.20%	77.80%	94.30%
		Model 2	0.946 (0.847~0.989)	0.326	>0.2421	87.50%	100.00%	100.00%	94.90%
		Model 3	0.971 (0.884~0.998)	0.088	>0.2401	87.50%	100.00%	100.00%	94.90%
	HCM vs non-HCM	Model 1	0.765 (0.629~0.871)	reference	>0.2447	90.00%	60.60%	58.10%	90.90%
		Model 2	0.855 (0.731~0.936)	0.179	>0.2537	95.00%	72.70%	67.90%	96.00%
		Model 3	0.885 (0.767~0.956)	0.067	>0.2488	90.00%	72.70%	66.70%	92.30%
	HHD vs non-HHD	Model 1	0.786 (0.652~0.887)	reference	>0.2170	76.50%	72.20%	56.50%	86.70%
		Model 2	0.820 (0.691~0.912)	*0.575*	>0.1947	94.10%	58.30%	51.60%	95.50%
		Model 3	0.848 (0.723~0.932)	*0.266*	>0.1809	94.10%	69.40%	59.30%	96.20%

4. Supplemental Figures

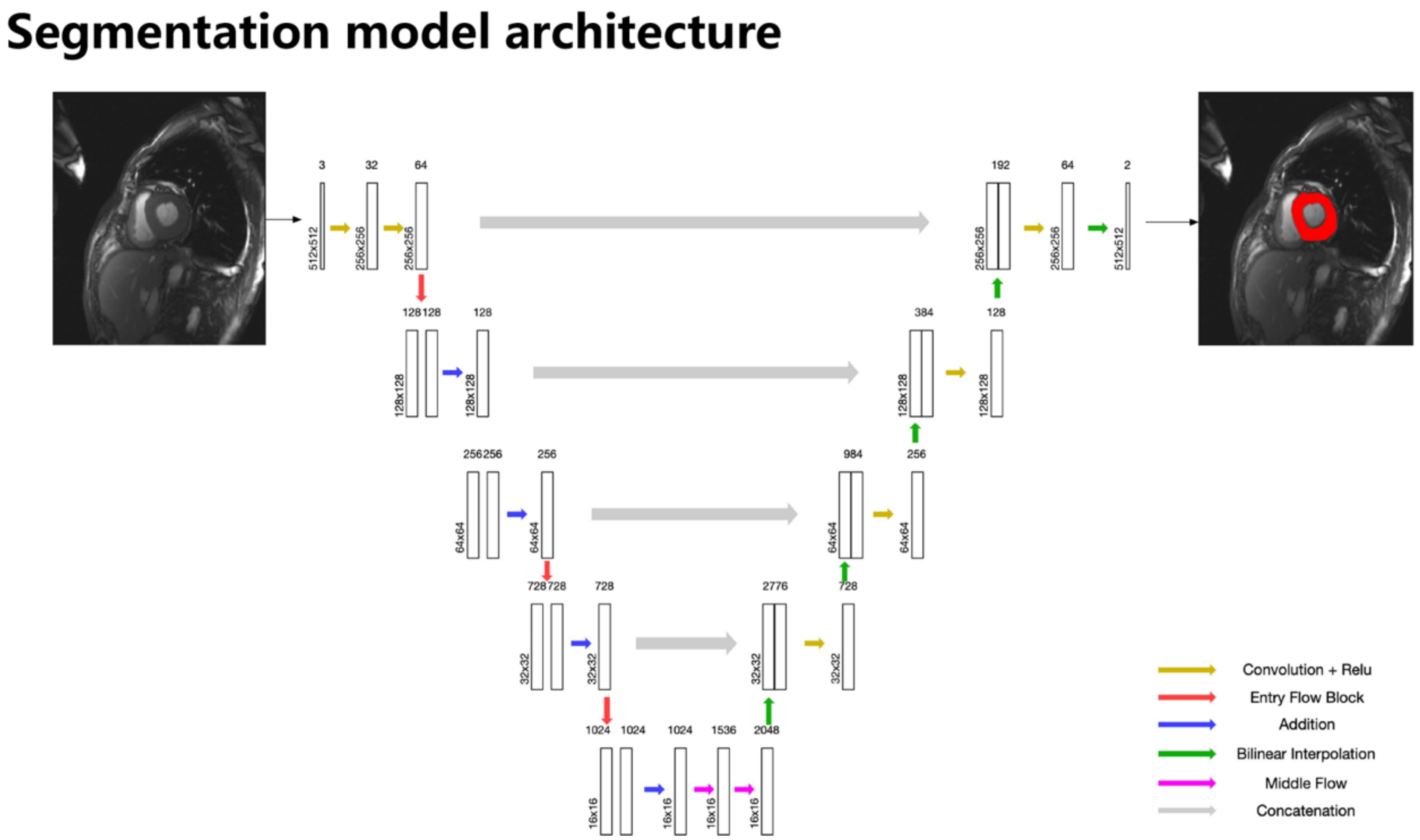

Supplemental Figure 1. Conceptual architecture of the Res-Unet model.

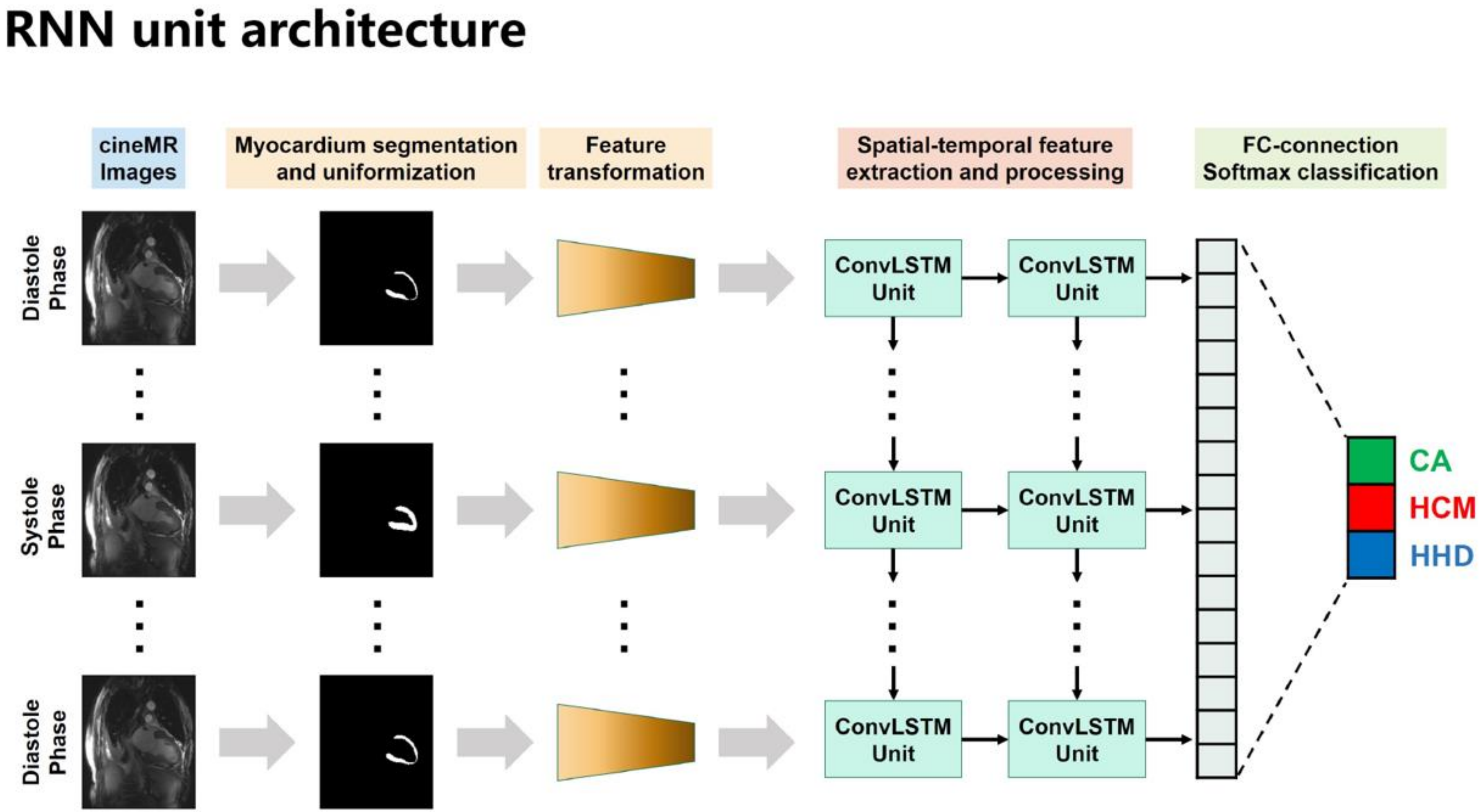

Supplemental Figure 2. The representation of model architecture for the single-channel RNN.

HCM, hypertrophic cardiomyopathy, CA, cardiac amyloidosis, HHD, hypertensive heart disease.

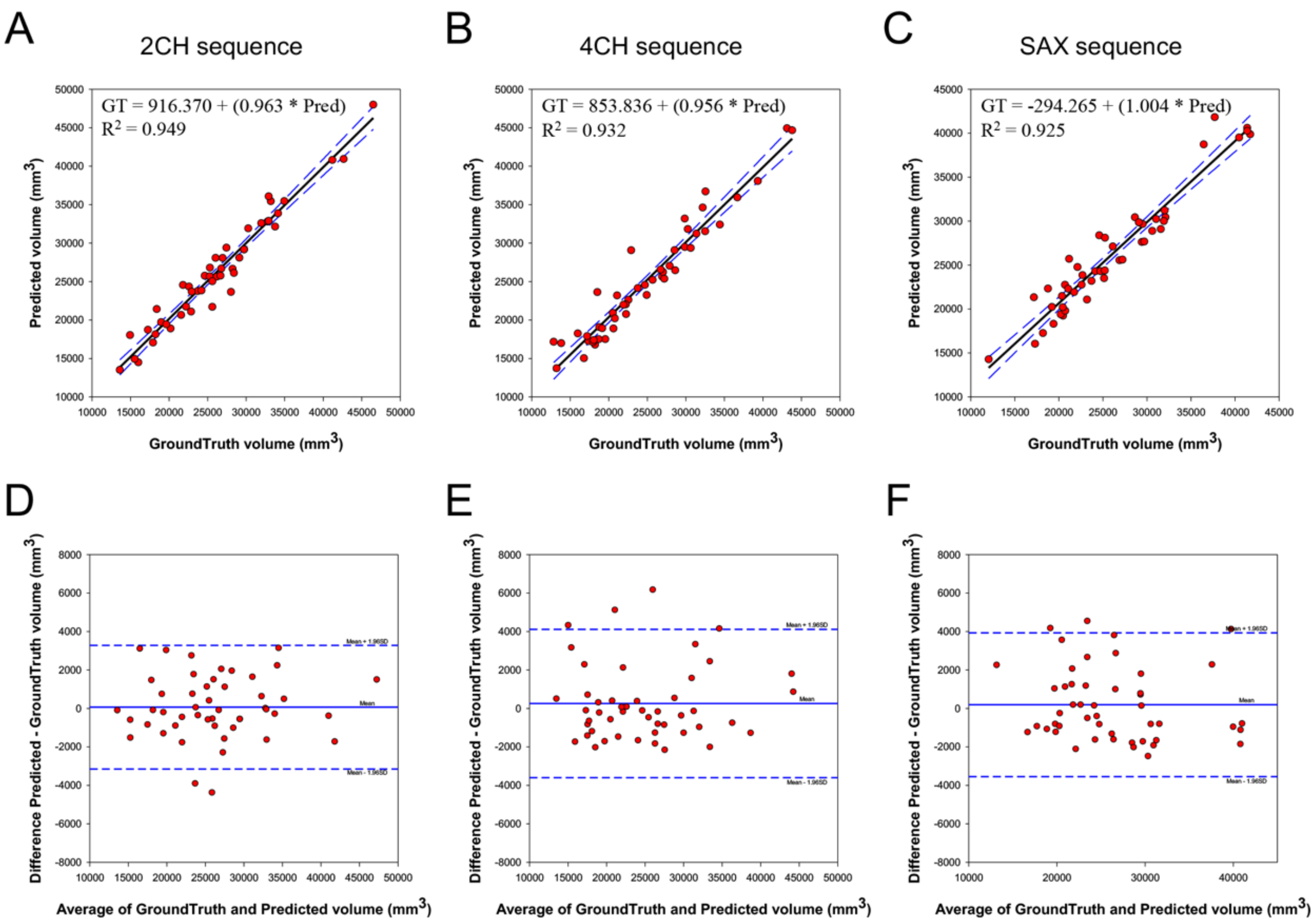

Supplemental Figure 3. Scatterplots and Bland-Altman plots of automated segmentation and manually labeled myocardium volumes in per-case analysis in the validation dataset. Correlation coefficient (R) and Bland-Altman analysis demonstrated good performance of the Res-Unet model for the 2CH sequence (A and D), 4CH sequence (B and E) and SAX sequence (C and F) cine MR images.

2CH, two-chamber, 4CH, four-chamber, SAX, short axis.

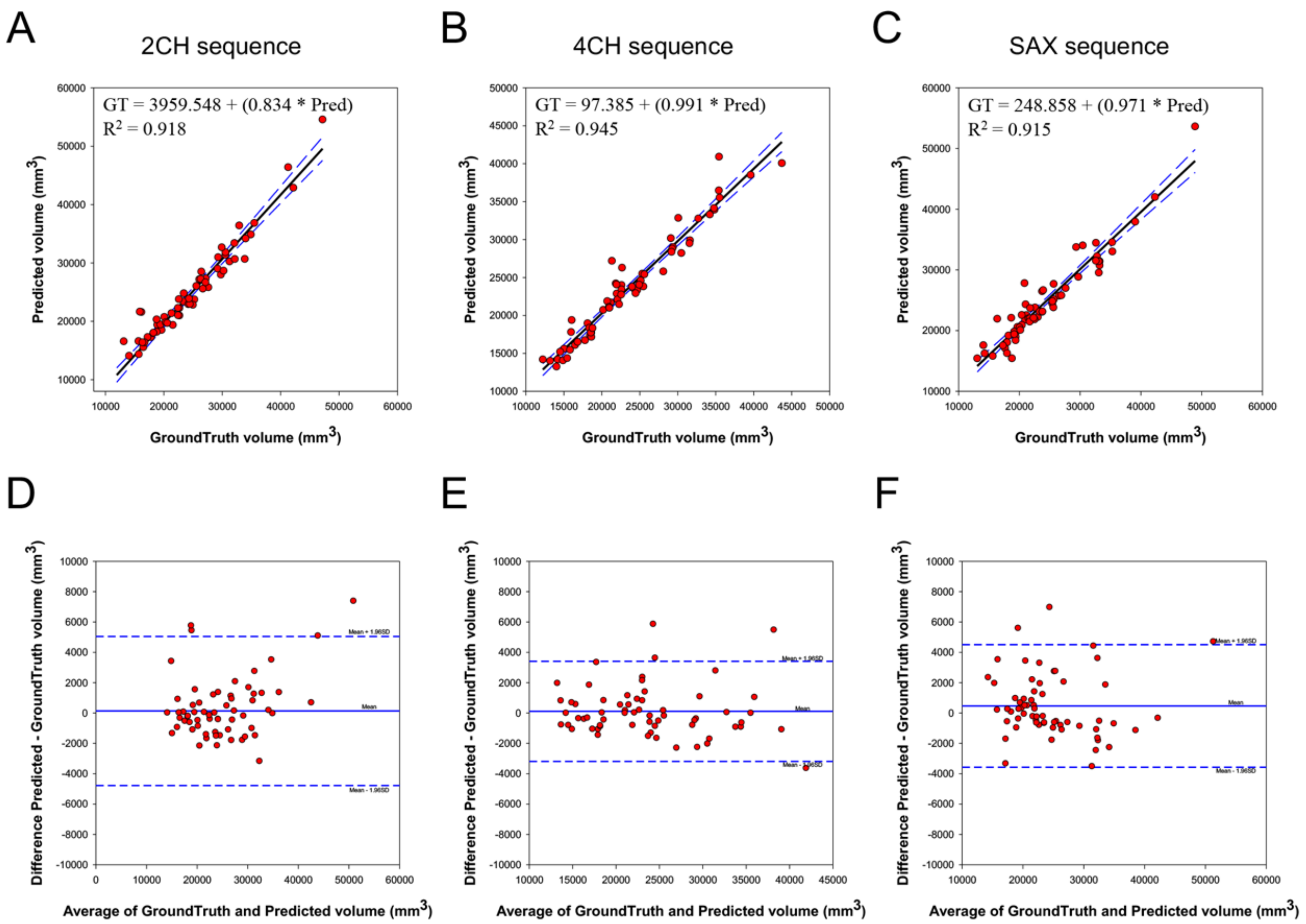

Supplemental Figure 4. Scatterplots and Bland-Altman plots of automated segmentation and manually labeled myocardium volumes in per-case analysis in the internal test dataset. Correlation coefficient (R) and Bland-Altman analysis demonstrated good performance of the Res-Unet model for the 2CH sequence (A and D), 4CH sequence (B and E) and SAX sequence (C and F) cine MR images.

2CH, two-chamber, 4CH, four-chamber, SAX, short axis.

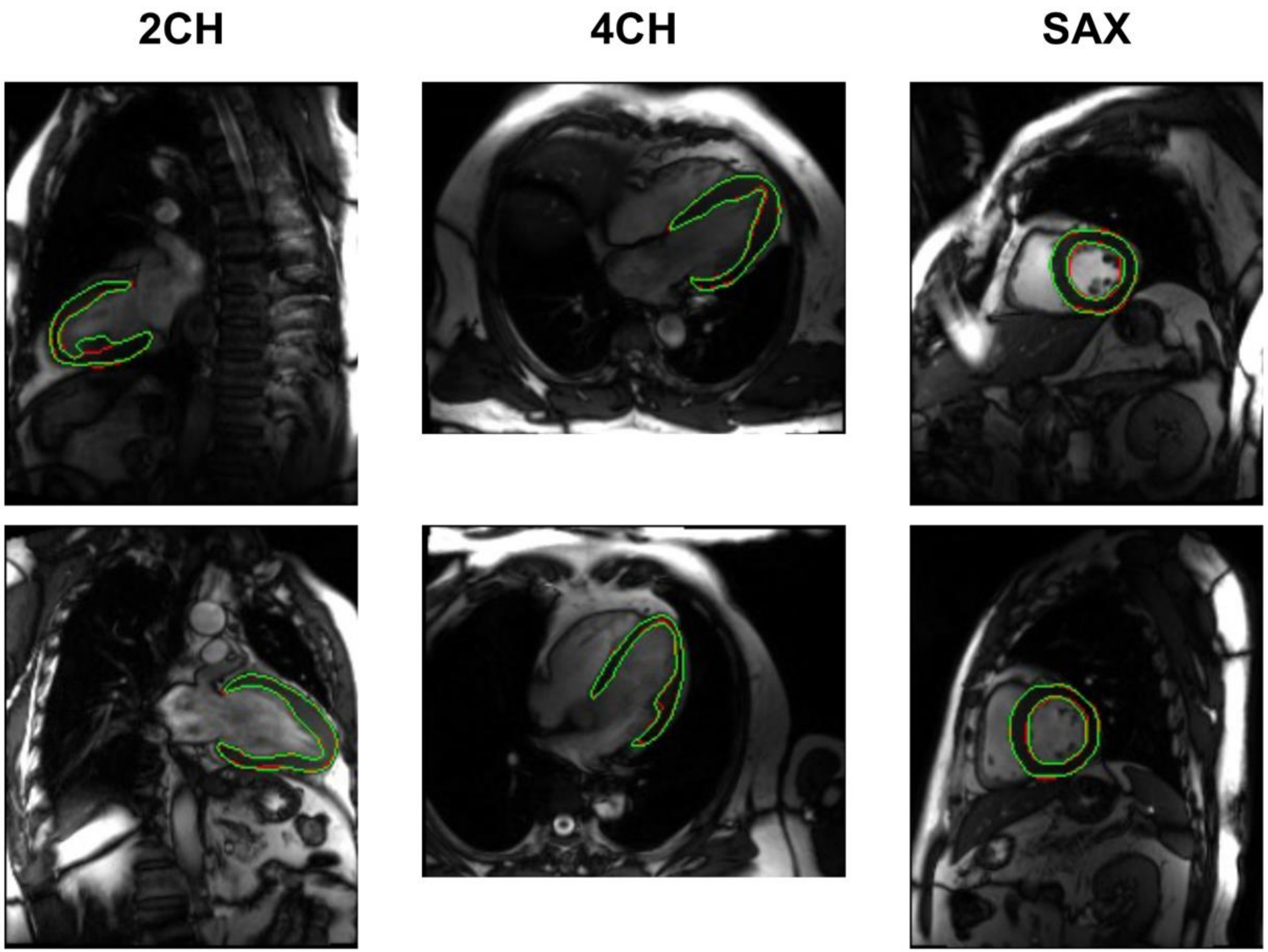

Supplemental Figure 5. Examples of the automatedly segmented 2CH (left), 4CH (middle) and SAX (right) cine MR images. Red lines were the boundary obtained with manual segmentation, and green lines indicated the area generated by the proposed method.

2CH, two-chamber, 4CH, four-chamber, SAX, short axis.

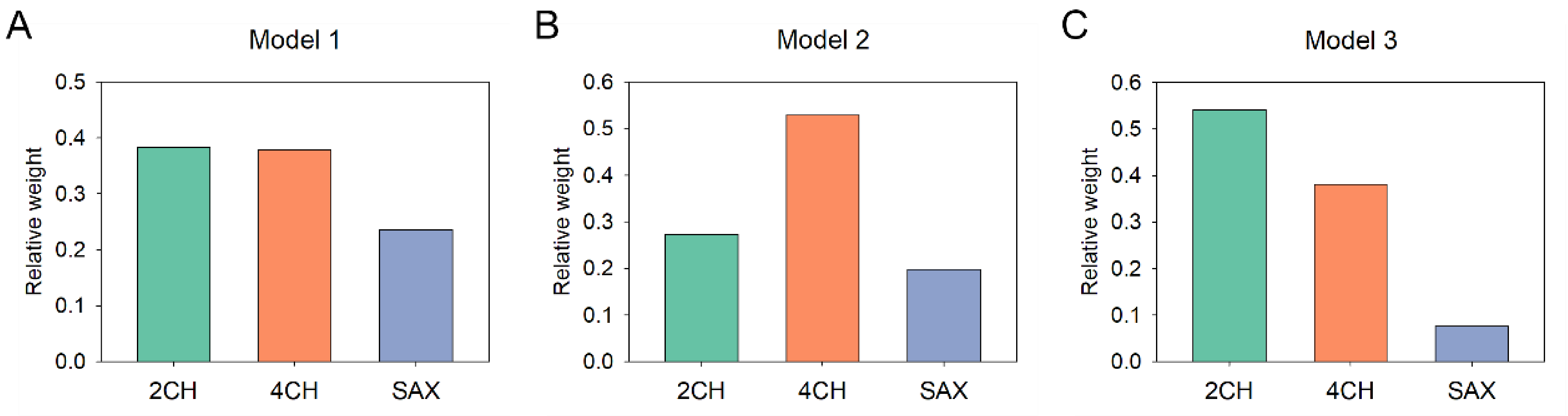

Supplemental Figure 6. Weight contribution analysis of the SVM model. The relative weights of the input image derived from 2CH, 4CH and SAX sequences of cine MR images were compared in Model 1 (A), Model 2 (B) and Model 3 (C), respectively. Higher weights indicated higher attribution to the final diagnosis.

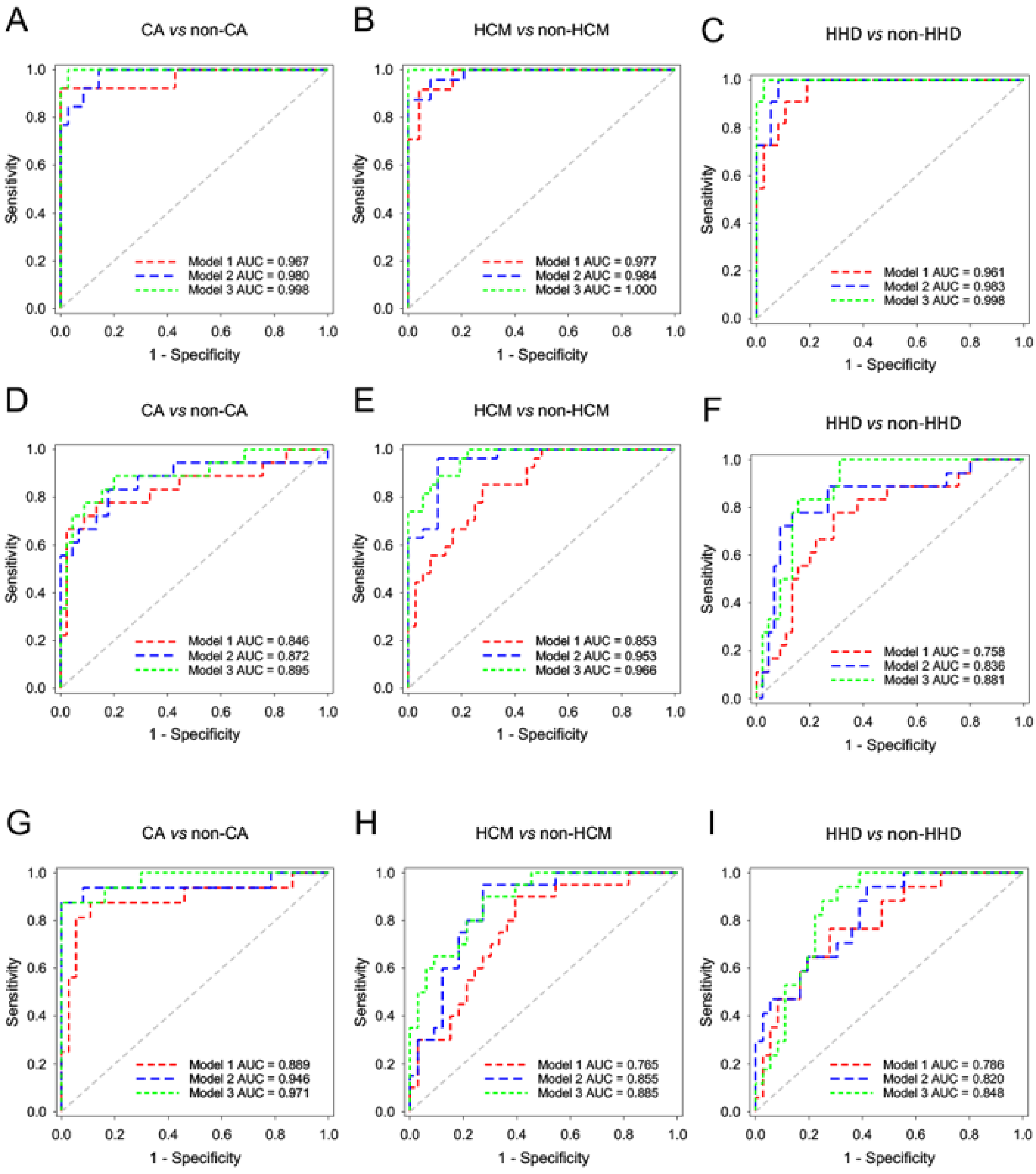

Supplemental Figure 7. ROC analysis for binary classification in the validation dataset (A-C), the internal test dataset (D-F) and the external test dataset (G-I).

HCM, hypertrophic cardiomyopathy, CA, cardiac amyloidosis, HHD, hypertensive heart disease, AUC, area under the curve.
